# Enhanced synaptic protein visualization by multicolor super-resolution expansion microscopy

**DOI:** 10.1117/1.NPh.10.4.044412

**Published:** 2023-10-25

**Authors:** Janna Eilts, Sebastian Reinhard, Nikolas Michetschläger, Christian Werner, Markus Sauer

**Affiliations:** a University of Würzburg, Department of Biotechnology and Biophysics, Biocenter, Würzburg, Germany; b University of Würzburg, Rudolf Virchow Center, Research Center for Integrative and Translational Bioimaging, Würzburg, Germany

**Keywords:** expansion microscopy, super-resolution microscopy, synaptic proteins, multicolor imaging, structured illumination microscopy, 10-fold robust expansion microscopy

## Abstract

**Significance:**

Understanding the organization of biomolecules into complexes and their dynamics is crucial for comprehending cellular functions and dysfunctions, particularly in neuronal networks connected by synapses. In the last two decades, various powerful super-resolution (SR) microscopy techniques have been developed that produced stunning images of synapses and their molecular organization. However, current SR microscopy methods do not permit multicolor fluorescence imaging with 20 to 30 nm spatial resolution.

**Aim:**

We developed a method that enables 4-color fluorescence imaging of synaptic proteins in neurons with 20 to 30 nm lateral resolution.

**Approach:**

We used post-expansion immunolabeling of eightfold expanded hippocampal neurons in combination with Airyscan and structured illumination microscopy (SIM).

**Results:**

We demonstrate that post-expansion immunolabeling of approximately eightfold expanded hippocampal neurons enables efficient labeling of synaptic proteins in crowded compartments with minimal linkage error and enables in combination with Airyscan and SIM four-color three-dimensional fluorescence imaging with 20 to 30 nm lateral resolution. Using immunolabeling of Synaptobrevin 2 as an efficient marker of the vesicle pool allowed us to identify individual synaptic vesicles colocalized with Rab3-interacting molecule 1 and 2 (RIM1/2), a marker of pre-synaptic fusion sites.

**Conclusions:**

Our optimized expansion microscopy approach improves the visualization and location of pre- and post-synaptic proteins and can thus provide invaluable insights into the spatial organization of proteins at synapses.

## 1 Introduction

The unique combination of molecule-specific labeling, minimal perturbation of biological complexes, and three-dimensional (3D) imaging makes fluorescence microscopy the most popular imaging modality in cell biology. To understand how a cell works and which mechanisms occur in the case of a dysfunction or disease, it is essential to understand how biomolecules are organized into complexes and the dynamics of this organization. This becomes especially apparent when we aim to visualize complex neuronal networks in brains connected by synapses. Synaptic transmission, the biological process by which a neuron communicates with a target cell across a synapse, relies on spatially and temporally coordinated multistep processes that require the precise organization of proteins. In the case of chemical synapses, transmission involves the release of a neurotransmitter from the pre-synaptic neuron, and neurotransmitter binding to specific post-synaptic receptors.^1^^,^^2^ However, despite their relevance for efficient transmission, knowledge about the molecular organization of proteins at synapses and their interplay remains limited. This is mainly due to the fact that major components of synapses are too small to be resolved by standard fluorescence microscopy, which is restricted to ∼200  nm by the diffraction barrier. For example, synaptic vesicles, key elements for transmitter cargo, exhibit diameters of 40 to 50 nm and release their transmitters into the synaptic cleft with a width of 20 to 30 nm where they finally bind to postsynaptic receptors.^3^^–^^5^ Therefore, electron microscopy was for decades the only method capable of resolving the nanoscale characteristics of synapses.^6^^,^^7^

In the last decade, super-resolution (SR) microscopy has evolved into a powerful method for sub-diffraction resolution fluorescence imaging of cells and structural investigations of cellular organelles.^8^ SR microscopy methods can now provide a spatial resolution that is well below the diffraction limit of light microscopy, enabling invaluable insights into the spatial organization of proteins at synapses.^5^ However, current SR measurements become error-prone below 20 nm.^9^^–^^12^ Even though the combination of sequential structured illumination with single-molecule localization microscopy enabled fluorescence imaging in cells approaching 10 nm resolution,^13^^,^^14^ these methods are highly complex requiring expert knowledge and sophisticated microscopes. In addition, they are limited to a few fluorophores that exhibit suited blinking performance in special photoswitching buffers or can be photoactivated by irradiation with ultraviolet light, which does not allow fluorescence imaging with more than 2 colors with 20 to 30 nm spatial resolution. In addition, the large number of proteins involved in synaptic signaling located within the small size of synaptic compartments creates a crowded situation that impedes fluorescence labeling at high densities required to resolve the molecular organization of synapses.^15^^,^^16^

Hence, the choice of labeling strategy is critical as it affects the achievable resolution via the linkage error (the displacement of the fluorophore from the target molecule) and the labeling density. Standard immunolabeling with primary and secondary antibodies, which is the method of choice for labeling of synaptic proteins, causes linkage errors between 10 and 20 nm.^11^^,^^17^ Furthermore, antibodies have to compete for epitope accessibility of densely packed synaptic proteins. Fab fragments and nanobodies are smaller than antibodies and therefore are good alternatives but efficient nanobodies are not available for every synaptic protein and even a size of only a few nanometers hampers high density labeling required to achieve a structural resolution of 20 to 30 nm.^15^^,^^16^ Here, we show how post-expansion immunolabeling of approximately eightfold expanded hippocampal neurons enables high density labeling of synaptic proteins and in combination with Airyscan and structured illumination microscopy (SIM) 3D fluorescence imaging of up to 4 target molecules with 20 to 30 nm lateral resolution.

## 2 Materials and Methods

### 2.1 Preparation of Hippocampal Mouse Neurons

Primary hippocampal neurons were prepared from E18 C57BL/6 mice (approved by Bavarian state authorities). The hippocampal tissue was digested in 0.05% trypsin Ethylenediaminetetraacetic acid (ThermoFisher 25300054) at 37°C for 15 min and washed twice in Hanks’ balanced salt solution (HBSS) consisting of 10× HBSS (ThermoFisher, 88284), 250  μl gentamycin (Sigma, G1397-10ML), 3.5 ml 1 M 4-(2-hydroxyethyl)-1-piperazineethanesulfonic acid (Sigma, H0887), filled up to 500 ml total volume using ${\mathrm{ddH}}_{2}O$. For trituration with different pipette pore sizes, the hippocampi were placed in neurobasal culture medium consisting of 100 ml neurobasal (ThermoFisher, A3582901), 1 ml glutamax (ThermoFisher, 35050061), 50  μl gentamycin, and 2% B27 plus serum free supplement (ThermoFisher, A3582801). Cells were seeded on 12 mm poly-D-lysine (PDL; Sigma, P6407) coated coverslips [1  mg/ml for 1 h at room temperature (RT), washed twice with ${\mathrm{ddH}}_{2}O$]. In total, 40,000 neurons were plated on each coverslip and incubated in neurobasal medium at 37°C, 5% ${\mathrm{CO}}_{2}$ for 14 days. Fifty percent of medium was replaced weekly. Primary neurons were fixed by applying 4% methanol-free paraformaldehyde (ThermoFisher, 289036) for 15 min at RT and washing twice with 1× phosphate-buffered saline (PBS) (Sigma, D1408-500ML).

### 2.2 Antibodies and N-hydroxysuccinimidyl-Dye Conjugation

Manufacturers and working concentrations of all primary and secondary antibodies are listed in Table 1. Goat anti rabbit secondary antibody (Invitrogen, 31212) was conjugated to NHS-ATTO643 (ATTO-TEC, AD 643-31) using Zeba™ Spin Desalting Columns 40K MWCO (ThermoFisher, 87766). Coupling was performed according to instructions in the manufacturer’s manual in 100 mM ${\mathrm{NaHCO}}_{3}$ (ThermoFisher, 144-55-8) with sevenfold molar excess of the NHS-dye. During 3 h of incubation at RT in the dark, the NHS-ester reaction proceeded. Afterward, the labeled antibodies were purified with Zeba™ Spin Desalting Columns in 0.02% ${\mathrm{NaN}}_{3}$ (Sigma, S-8032) in 1× PBS to get rid of free dye. Antibody concentration and degree of labeling were calculated after measuring absorption at 280 and 643 nm at a nanophotometer (Implen).

**Table 1:** Compilation of used antibodies.

Antibody	Host	Working concentration (μg/ml)	Product no.
*Primary antibodies*			
Synaptobrevin 2	Mouse	10	104 202 SYSY
RIM1/2	Guinea pig	10	140 205 SYSY
Homer1	Rabbit	5	160 003 SYSY
VGlut1	Guinea pig	5	135 304 SYSY
Munc13-1	Rabbit	5	126 103 SYSY
Neurofilament-L	Mouse	10	171 011 SYSY
*Secondary antibodies*			
Mouse AF488	Goat	20	A11017 Invitrogen
Guinea pig AF488	Goat	10	106-546-003 Jackson
Guinea pig CF568	Donkey	10	SAB4600469 Sigma
Rabbit CF568	Goat	10	SAB4600400 Sigma
Rabbit ATTO643	Goat	10	Self-labeled

### 2.3 Pre-Gelation Immunostaining

For comparison to post-expansion labeling, neurons were immunostained before gelation. Fixed cells were permeabilized for 10 min with 0.1% triton™ X-100 (ThermoFisher, 28314) and washed 3 times with 1× PBS. After blocking with 5% bovine serum albumin (BSA, Sigma, A7030) for 30 min, neurons were incubated with primary antibodies for 2 h in 5% BSA. Following three washing steps with 1× PBS, secondary antibodies were added for another 2 h in 5% BSA and washed again 3 times with 1× PBS. Same concentrations of the respective antibodies were selected for pre- and post-expansion staining to enable comparability.

## 3 Crosslinking, Gelation, and Denaturation

Pre-stained neurons or untreated fixed neurons were crosslinked with 4% formaldehyde (Sigma, F8775-25ML) and 30% acrylamide (Sigma, A4058) for 18 h at 37°C. For the gelation, 10-fold robust expansion microscopy (TREx) monomer solution^18^ consisting of 1.1 M sodium acrylate (Sigma, 408220), 2.0 M acrylamide, 0.009% N,N′-methylenebisacrylamide (Sigma, M1533), and 1× PBS was prepared, aliquoted, and stored at −20°C. Freshly thawed monomer solution was mixed with 0.15% N,N,N′,N′-tetramethylethylenediamine (Sigma, T7024) and 0.15% ammonium persulfate (Sigma, A3678) shortly before gelation. A drop of 60  μl gel solution was pipetted on a piece of parafilm inside a gelation box on ice, and a coverslip was flipped onto it with the cell side facing down. The gels polymerized in a humidified chamber for 3 h at RT. To homogenize the solidified gels, denaturation buffer consisting of 200 mM sodium dodecyl sulfate (Invitrogen, AM9820), 300 mM NaCl (Sigma, S5886), and 50 mM tris-(hydroxymethyl)-aminomethan (Sigma, 1.08382) was prepared and adjusted to pH 9. After the buffer was pre-warmed to 100°C, gels were denatured for 1 h at 100°C. For post expansion, immunostaining gels were washed 3 times for 30 min in 14 cm petri dishes with 1× PBS.

### 3.1 Post-Gelation Immuno- and NHS-Staining

After washing out denaturation buffer with 1× PBS, gels were post-labeled in an approximately threefold expanded state. They were incubated with primary antibodies in 5% BSA overnight on a spinning wheel at RT. After washing 3 times for 15 min with 0.1% TWEEN (Sigma, P9416) in 1× PBS (PBST), gels were incubated with secondary antibodies for 3 h at 37°C. Three more washing steps with 0.1% PBST were applied prior to whole proteome labeling via NHS-dyes. For this, gels were incubated for 1.5 h with 40  μg/ml NHS-CF405M (Biotium, 92111) dissolved in 100 mM ${\mathrm{NaHCO}}_{3}$ in 1× PBS. Finally, gels were fully expanded by washing 6 to 7 times in a large volume of ${\mathrm{ddH}}_{2}O$.

### 3.2 Coating of Imaging Chambers

To reduce gel drift while imaging, one-well chambered cover glasses (Cellvis, C1-1.5H-N) were coated with 0.1  mg/ml PDL for 1 h at 37°C. Expanded gels were cut and placed in the chamber with the cell side facing the cover slip.

### 3.3 Image Acquisition and Processing

Airyscan images were acquired at a LSM 900 with Airyscan 2 (Zeiss) in SR imaging mode using a C-Apochromat 40×/1.2 numerical aperture (NA) water-immersion objective (Zeiss). Suitable excitation wavelengths and filter settings for the respective dyes were selected via the integrated dye presets in the ZEN 2 blue software (Zeiss, version 3.5). All images were processed in the standard strength mode of 3D Airyscan processing. $\mathrm{SI}M^{2}$ images were recorded at an Elyra 7 (Zeiss) equipped with a C-Apochromat 63×/1.2 NA water objective (Zeiss) and high-reflectivity (HR) Diode 405 nm (50 mW), HR Diode 488 nm (500 mW), HR diode-pumped solid state 561 nm (500 mW), and HR Diode 642 nm (500 mW). Emission light was directed onto 2 scientific complementary metal-oxide-semiconductor PCO Edge 4.2 M cameras using a beamsplitter (SBS LP 560). In addition, filtersets band pass (BP) 570-620 + long pass (LP) 655 and BP 420-480 + BP 495-550 and a laser blocking filter LBF 405/488/561/642 were selected. $\mathrm{SI}M^{2}$ processing was performed with the Zeiss ZEN 3.0 SR FP2 (black) software with either 8 (for 4-color images) or 12 iterations (for pre- versus post-staining). During $\mathrm{SI}M^{2}$ processing, a channel alignment was performed to compensate for chromatic aberration. Therefore, 100 nm Tetraspecks™ (Invitrogen, T7279) were diluted 1:1000 ${\mathrm{ddH}}_{2}O$ and incubated with an unstained gel. Z-stacks were recorded and processed with the same settings as the respective images, and an alignment matrix was generated. In all images, brightness and contrast were adjusted linearly with the help of the software Fiji (version 2.9.0/1.53t).^19^ 3D images were created in the software Imaris (Oxford Instruments, version 9.2.1) applying a maximum intensity projection or with the option normal shading for improved volume visualization.

### 3.4 Expansion Factor Determination

The expansion factor was determined by comparing the pre- and post-expanded gel air−water boundary or by the distances between landmarks in the pre- and post-expanded cells. Furthermore, we compared cross-sectional profiles of pre-expansion immunolabeled neurofilament L^18^ in primary neurons before and after expansion to determine a structural expansion factor of 8.39 (Fig. S1 in the Supplementary Material). For this purpose, gels were imaged with a Nikon confocal AX setup. This system allows for image capture with a field of view measuring 8192×8192  pixels, significantly facilitating the identification of the same area in the gel before and after expansion. The microscope is equipped with an AX R galvano scan head and a 2K resonant scanner. For confocal imaging, a DUX-VB detector unit containing four gallium arsenide phosphide photomultiplier tubes was used. To image the non-expanded sample, a Plan apochromat (APO) 60×/1.24 oil immersion objective was employed while a Plan APO 60×/1.27 water immersion objective was utilized for the expanded sample. Nikon’s NIS-Elements C software was used to select the appropriate excitation wavelength and filters via the integrated dye preset for AF488. To determine a structural expansion factor, we registered the same structure with two different transformations. The initial similarity transform covers degrees of freedom that occurs under isotropic expansion and rearrangement under the microscope, such as rotation, translation in x and y, and scale. Here, the scale parameter determines the structural expansion factor. To visualize nonlinear distortions, we further perform an affine transform, including additional degrees of freedom. The difference between similarity and affine transform is depicted in a distortion map.

### 3.5 Quantification of Particles

To determine the number of Vglut1 clusters detected and thus obtain an indication of the reduced linkage error, a particle analysis was carried out. Based on recordings of Vglut1 pre- and post-expansion labeled gels, distinguishable particles were quantified using Fiji. After background subtraction, an auto threshold was set using the “default” method. Thresholding yielded a mask that was segmented with “watershed” algorithm, and the resulting particles were counted with “analyze particles.” With the help of the software origin, data were plotted and tested for significance using a two-sample t-test with equal variance (Shapiro–Wilk: $p_{\mathrm{pre}}=0.18$, $p_{\text{post}}=0.11$; Levenes: p=0.78). The mean value of the number of post-expansion labeled particles is significantly larger than the mean value of pre-expansion labeled particles (p=0.017).

## 4 Results

Currently, immunolabeling represents the method of choice for visualization of synaptic proteins in pre- and post-synaptic compartments but the size of antibodies prevents dense labeling with minimal linkage error required for reliable multicolor fluorescence imaging with 20 to 30 nm nanometer spatial resolution. This labeling barrier thus determines the achievable structural resolution independent of the resolution capacity of the used SR microscopy method. Fortunately, there is a solution to this problem that enables improved labeling of synaptic proteins in dense compartments with reduced linkage error and multicolor super-resolved fluorescence imaging on standard microscopes. The approach, termed expansion microscopy, uses the physical expansion of the cellular structure by linking proteins into a dense, cross-linked network of a swellable polyelectrolyte hydrogel that expands after enzymatic digestion or denaturation in water.

Since its first introduction by Chen et al.,^20^ expansion microscopy has shown impressive results, including the magnified visualization of proteins, lipids, and nucleic acids with fluorescent proteins, antibodies, functionalized sphingolipids, and oligonucleotides, respectively, in cells, tissues, and human clinical specimen.^21^^,^^22^ It is possible to expand samples in series enabling gel expansion factors of 20× and higher^23^ but such high expansion factors reduce the labeling density and consequently the signal-to-noise ratio thus requiring highly sensitive imaging methods. Airyscan confocal and SIM provide only moderate resolution improvement factors of ∼1.4^24^ and ∼2.0^8^ translating into lateral resolutions of up to ∼140 and 100 nm, respectively, but they enable 4-color fluorescence imaging with various fluorophores spanning the entire visible spectrum. Hence, expansion factors of 8 to 10× allow us to visualize the distribution of up to 4 proteins simultaneously in synapses at lateral resolutions of at least 20 to 30 nm.

**Fig. 1:**
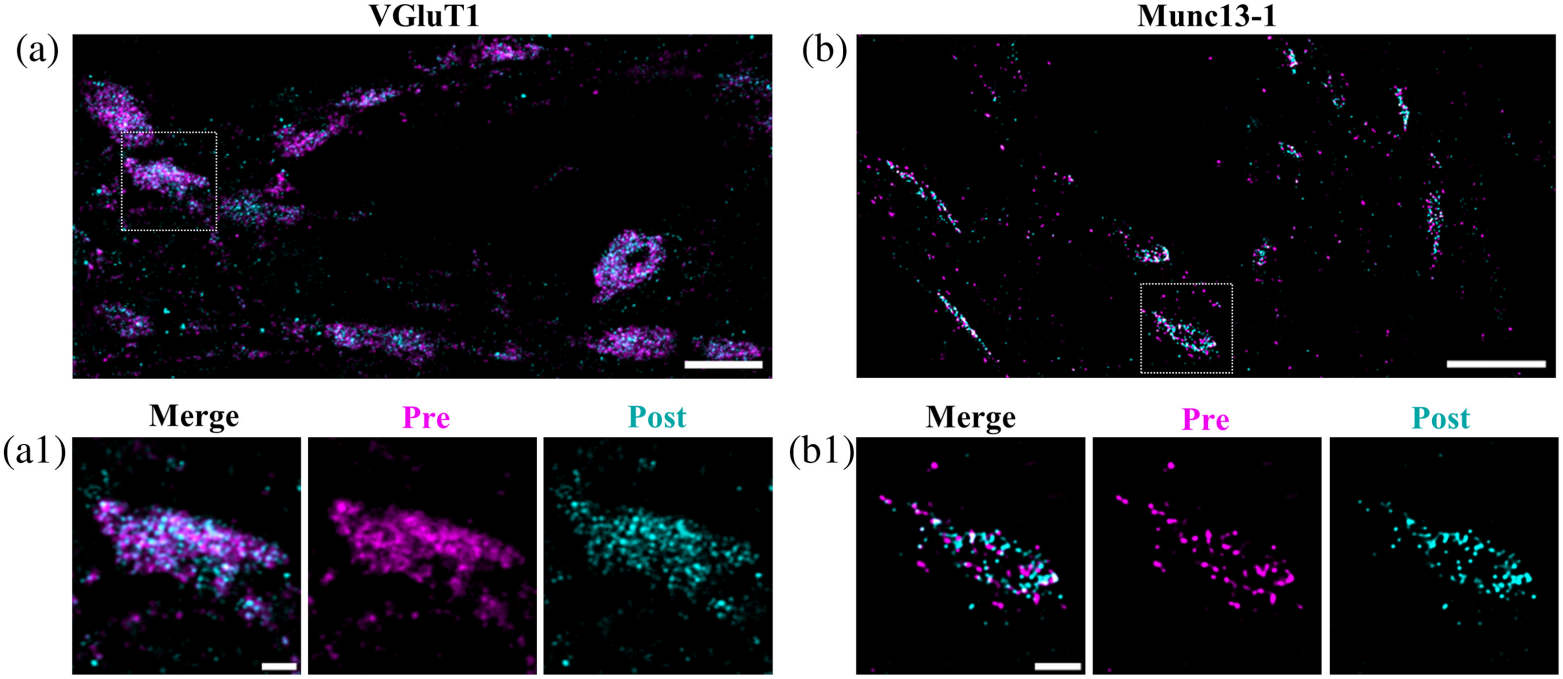
Post-expansion immunostaining enables efficient labeling of proteins in crowded synaptic compartments. (a) Airyscan image (maximum projection of z-stack) of approximately eightfold expanded hippocampal neurons stained pre- and post-gelation with the same primary VGluT1 antibody. The secondary antibodies were labeled with CF568 (pre-gelation, magenta) and AF488 (post-gelation, cyan). Scale bar 10  μm. (a1) Magnified images (single z-stack slice) of the marked pre-synaptic vesicle bouton from panel (a) show that post-expansion immunolabeling resolves more synaptic details. Scale bars 2  μm. (b) $\mathrm{SI}M^{2}$ image (maximum projection of z-stack) of approximately eightfold expanded hippocampal neurons stained pre- and post-gelation with the same primary Munc13-1 antibody. The secondary antibodies were labeled with ATTO643 (pre-gelation, magenta) and CF568 (post-gelation, cyan). Scale bar 10  μm. (b1) Magnified images (single z-stack slice) of the marked active zone show that post-expansion immunolabeling resolves more synaptic details and exhibits a smaller linkage error. Scale bars 2  μm. Scale bars of expanded hydrogels are shown.

To compare the labeling densities of pre- and post-expansion immunostaining, we first labeled the pre-synaptic proteins VGluT1 and Munc13-1 in hippocampal neurons with identical primary and secondary antibodies before and after expansion (Fig. 1).^25^^–^^28^ After the first pre-expansion immunolabeling step, we used TREx^29^ applying formaldehyde and acrylamide as an anchoring molecule for proteins followed by high-temperature denaturation after gelation. Proteinase K digestion was omitted to preserve protein epitopes. After initial approximately threefold expansion in PBS, synaptic proteins were immunostained once again with the same primary and secondary antibodies labeled with a different fluorophore overnight. After washing, the samples were placed in ${\mathrm{ddH}}_{2}O$ to fully expand approximately eightfold as determined from the comparison of the hydrogel size before and after expansion and the cross-sectional profiles of pre-expansion immunolabeled neurofilament L^18^ in primary neurons before and after expansion using the same protocol (Fig. S1 in the Supplementary Material). The expansion factor is slightly lower than in the original TREx protocol most likely due to the protease-free treatment. Furthermore, it has to be considered that expansion factors may not be perfectly homogenous throughout the sample and can vary for different cellular organelles.^30^ The resulting Airyscan microscopy and SIM images clearly demonstrate that our protocol exhibits excellent protein retention, i.e., protein epitopes survive the denaturation, gelation, and expansion step in the absence of proteinase K, i.e., synaptic proteins can be efficiently immunostained post-expansion (Fig. 1).

The slightly higher labeling densities measured for post-expansion immunolabeling corroborate that additional protein epitopes become accessible for antibody labeling after threefold expansion [compare pre- and post-expansion labeled images in Figs. 1(a1) and 1(b1)]. An additional benefit of post-expansion labeling is a reduction of the linkage error proportional to the expansion factor compared to pre-expansion labeling. In our experiments, the immunolabeling linkage error of 17.5 nm defined by the primary and secondary antibodies^31^ decreases to only ∼2  nm in approximately eightfold expanded samples. Ultimately, the improved epitope accessibility and reduced linkage error of post-expansion immunolabeling facilitate the visualization of individual pre-synaptic vesicles (Fig. S2 in the Supplementary Material).

**Fig. 2:**
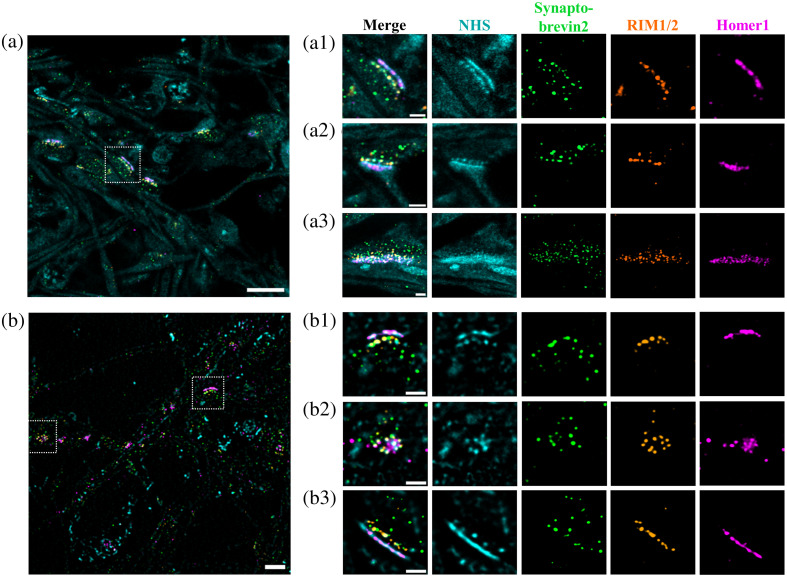
Synaptic protein organization visualized by four-color imaging of approximately eightfold expanded synapses. (a) Representative Airyscan overview image of hippocampal neurons using NHS-staining (cyan, CF405M) and immunostaining for Synaptobrevin2 (green, AF488), RIM1/2 (orange, CF568), and Homer1 (magenta, ATTO643). Scale bar 10  μm. (a1) Magnified side view images of a single synapse marked by the white rectangle in panel (a). Scale bars 2  μm. (a2, a3) Additional side view and slightly tilted (a3) synapse images. Scale bars 2  μm. (b) Representative SIM^2^ overview image with whole proteome NHS-staining (cyan, CF405M) and immunostaining for Synaptobrevin2 (green, AF488), RIM1/2 (orange, CF568), and Homer1 (magenta, ATTO643). Scale bar 10  μm. (b1, b2) Magnified images of single synapses marked by the white rectangles in panel (b) show a side and frontal view of a synapse. Scale bars 2  μm. (b3) Additional synapse side-view image from another overview image. Scale bars 2  μm. Scale bars of expanded hydrogels are shown (Video 1, MP4, 13.1 MB [URL: https://doi.org/10.1117/1.NPh.10.4.044412.s1]).

Next, we performed multicolor post-expansion immunolabeling of the postsynaptic density protein Homer1^32^ (ATTO643) and RIM1/2 (CF568), a key component of the pre-synapse that regulates vesicle docking and transmitter release efficiency at the active zone.^33^^,^^34^ In addition, we immunolabeled Synaptobrevin 2 (AF488), the major SNARE protein (soluble N-ethylmaleimide sensitive factor attachment protein receptor) with a high copy number on synaptic vesicles required for fast calcium-triggered synaptic-vesicle exocytosis and endocytosis.^35^^–^^38^ Finally, we labeled lysine residues of all synaptic proteins with CF405M-NHS to visualize local protein densities.^39^ Similar to correlative light and electron microscopy, this approach enables the localization of immunolabeled proteins in the cellular context provided by protein density. Airyscan microscopy and SIM images of eightfold expanded and post-expansion labeled hippocampal neurons clearly demonstrate that CF405M-NHS labeling enables identification of synapses because of the higher protein density (Fig. 2 and Fig. S3 in the Supplementary Material). Furthermore, Airyscan microscopy and SIM side view images of approximately eightfold expanded synapses show that synaptic clefts can be easily resolved confirming that the method achieves a lateral resolution of at least 20 to 30 nm (Fig. 2 and Fig. S3 in the Supplementary Material).

Closer inspection of the Homer1 and RIM1/2 signals reveals that the pre-synaptic as well as postsynaptic protein densities are not homogeneously distributed along the synaptic cleft (Fig. 2, Fig. S3 in the Supplementary Material, and Video 1). In particular, RIM1/2 appears to be compartmentalized in sub-synaptic domains (SSDs).^28^^,^^40^ Recently, it has been shown that postsynaptic SSDs at excitatory synapses are aligned with pre-synaptic SSDs, forming so-called trans-synaptic nanocolumns.^41^ Another SR microscopy study recently demonstrated the existence of trans-synaptic nanocolumns comprising SSDs of RIM1/2 at the pre-synapse and gephyrin, glycine receptors, and gamma-aminobutyric acid type A receptors at the postsynapse showing that this concept is conserved at inhibitory synapses.^42^ This led to the proposal that SSDs are functional units corroborating efficacy of neurotransmission and synaptic plasticity.^40^^,^^41^ Closer inspection of our expanded 2D and 3D four-color fluorescence images shows that synaptic vesicles close to the pre-synaptic membrane overlay with the RIM1/2 signals (Fig. 2, Fig. S3 in the Supplementary Material, and Video 1).

## 5 Discussion and Conclusions

Our data demonstrate that approximately eightfold expansion of neurons in combination with post-expansion immunolabeling enables 3D 4-color SR fluorescence imaging with 20 to 30 nm lateral resolution and minimal linkage error (Figs. 1 and 2, Figs. S2 and S3 in the Supplementary Material, and Video 1). The fact that we can resolve individual immunolabeled neurofilaments with a diameter of 10 nm^18^ in densely packed neurofilament bundles in neurons confirms that our method actually achieves at least 20 nm lateral resolution (Fig. S4 in the Supplementary Material). Such high resolutions and the ability to visualize three synaptic proteins simultaneously in context to the overall protein density outperforms approaches with higher expansion factors because they come along with higher dilution factors and consequently lower signal-to-noise ratios impeding high quality fluorescence imaging by confocal microscopy and SIM. Certainly, imaging of expanded neurons by single-molecule localization microscopy facilitates higher spatial resolutions but requires re-embedding of the expanded samples in a neutral polyacrylamide gel and remains limited to two-color imaging.^27^^,^^43^^,^^44^ Using immunolabeling of Synaptobrevin 2 as an efficient marker of the vesicle pool^45^^,^^46^ and four-color SR fluorescence imaging allowed us to identify individual synaptic vesicles colocalized with fusion sites (immunolabeling of RIM1/2) (Fig. 2, Fig. S3 in the Supplementary Material, and Video 1).^41^^,^^47^^,^^48^ Therefore, we expect that multicolor SR expansion microscopy in combination with post-expansion immunolabeling is ideally suited to address important open questions about the 3D molecular organization of synapses.

## Code, Data, and Materials Availability

The data are available from the corresponding authors upon reasonable request.
