# Supplementary material for: Enhanced synaptic protein visualization by multicolor super-resolution expansion microscopy
Source: Neurophotonics. 2023 Oct 25;10(4):044412. doi: 10.1117/1.NPh.10.4.044412 (PMC10599331; doi:10.1117/1.NPh.10.4.044412)
Supplement: Supplementary file 1 [file NPh_010_044412_SD001.pdf]

Supplementary Material

Enhanced synaptic protein visualization by multicolor super-resolution expansion microscopy

Janna Eilts[a], Sebastian Reinhard[a], Nikolas Michetschläger[a], Christian Werner[a], Markus Sauer[a,b,*]

[a]Department of Biotechnology and Biophysics, Biocenter, University of Würzburg, Am Hubland, 97074 Würzburg, Germany

[b]Rudolf Virchow Center, Research Center for Integrative and Translational Bioimaging, University of Würzburg, Josef-Schneider-Str. 2, 97080 Würzburg, Germany

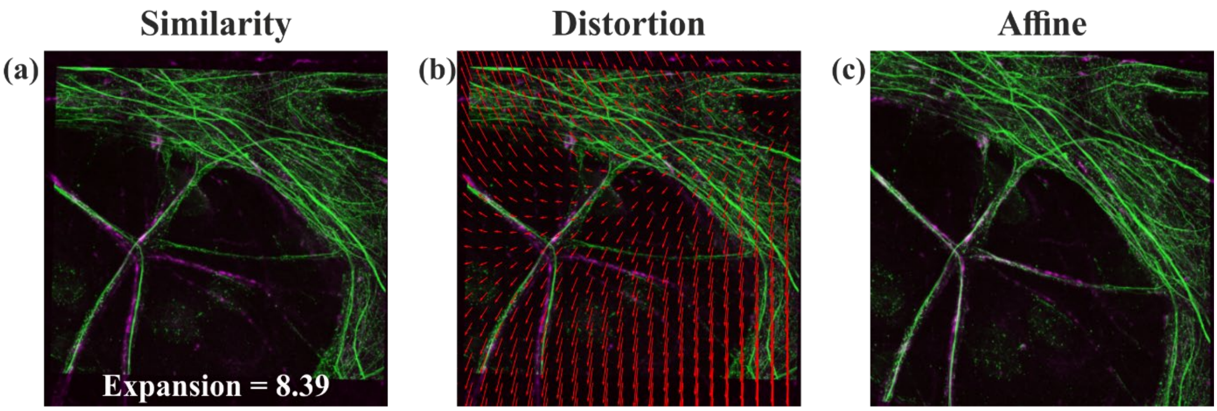

Figure S1. Determination of the structural expansion factor (a) Neurofilament L staining in primary neurons before (magenta) and after (green) expansion. The similarity transform encompasses the four degrees of freedom that occur during isotropic expansion (rotation, scaling, and translation in x and y) and yields a PCC of 0.17. **(b)** The vectorial shift from c to a required for nonlinear alignment is depicted by the distortion map. **(c)** An affine transform was used to further align the images. The images demonstrate a slightly improved PCC (0.30).

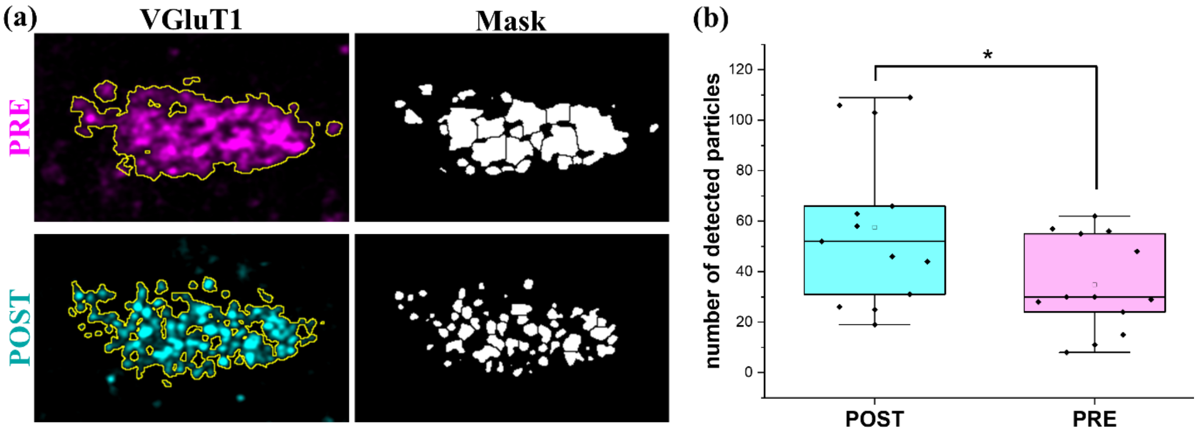

Figure S2. Quantification of detectable VGluT1 clusters indicates reduced linkage error by post-expansion labeling. (a) Representative 8-fold expanded presynaptic region of VGluT1 stained pre- (magenta) and post-expansion (cyan) with the corresponding mask obtained by particle analysis. **(b)** The mean number of distiguishable clusters by post-labeling is significantly higher (Two-sample t-test, $p = 0.017$, $n = 13$) than in pre-expansion labeling.

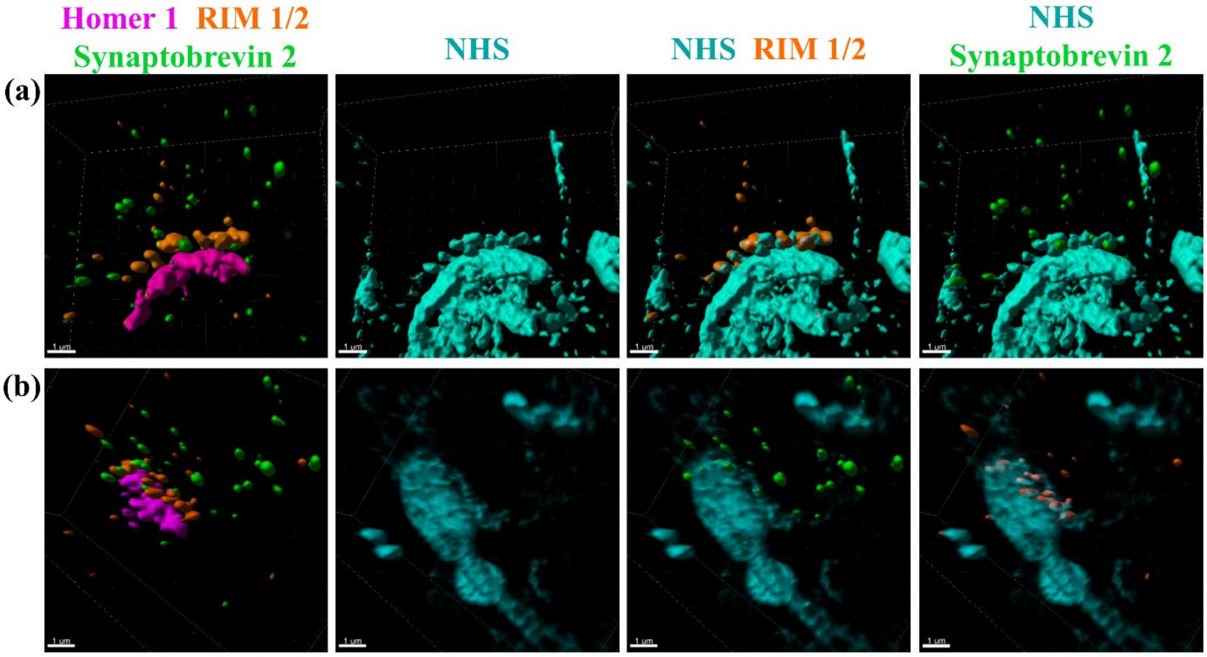

Figure S3. Representative 3D Airyscan images of 8-fold expanded synapses (see also Supplementary Video 1). The presynapse is post-expansion immunostained for RIM1/2 (orange) and Synaptobrevin2 (green). The postsynapse is marked by Homer1 (magenta) and synaptic context by NHS-staining (cyan). All channels are shown with the “Normal Shading” option from the software IMARIS except for NHS-channel in (b), which is displayed as Maximum Intensity Projection for improved visibility. Scale bars 1 µm (a, b)(expanded dimension).

Supplementary Video 1. 3D video of a synapse post-expansion immunostained for RIM1/2 (orange), Synaptobrevin 2 (green) and Homer1 (magenta).

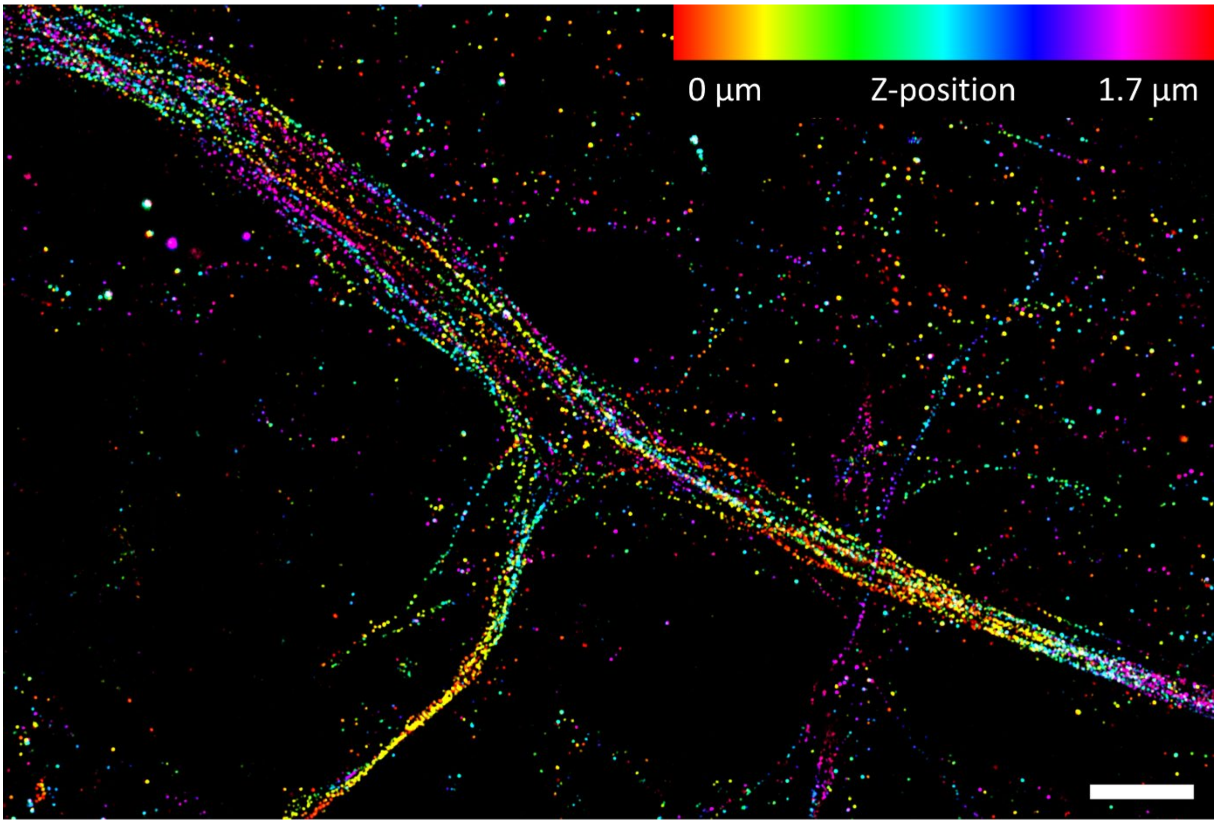

Figure S4. Color-coded maximum intensity projection of a 8-fold expanded neuron post-expansion stained for Neurofilament L and imaged with Airyscan. Partial distinctness of individual neurofilaments (with a known diameter of 10 nm)[19] confirms that the method achieves a lateral resolution of at least 20-30 nm. Scale bar 10 µm (expanded dimension).
